# A Self-Adaptive Dynamic Recognition Model for Fatigue Driving Based on Multi-Source Information and Two Levels of Fusion

**DOI:** 10.3390/s150924191

**Published:** 2015-09-18

**Authors:** Wei Sun, Xiaorui Zhang, Srinivas Peeta, Xiaozheng He, Yongfu Li, Senlai Zhu

**Affiliations:** 1 School of Information and Control, Nanjing University of Information Science & Technology, Nanjing 210044, China; 2 School of Computer and Software, Nanjing University of Information Science & Technology, Nanjing 210044, China; E-Mail: xrzhang@nuist.edu.cn; 3 School of Civil Engineering, Purdue University, West Lafayette, IN 47907, USA; E-Mail: peeta@purdue.edu; 4 The NEXTRANS Center, Purdue University, West Lafayette, IN 47906, USA; E-Mails: seanhe@purdue.edu (X.H.); liyongfu@cqupt.edu.cn (Y.L.); senlai@seu.edu.cn (S.Z.); 5 College of Automation, Chongqing University of Posts and Telecommunications, Chongqing 400065, China; 6 School of Transportation, Southeast University, Nanjing 210096, China

**Keywords:** fatigue driving, multi-source information, correlation analysis, fuzzy neural network, evidence theory

## Abstract

To improve the effectiveness and robustness of fatigue driving recognition, a self-adaptive dynamic recognition model is proposed that incorporates information from multiple sources and involves two sequential levels of fusion, constructed at the feature level and the decision level. Compared with existing models, the proposed model introduces a dynamic basic probability assignment (BPA) to the decision-level fusion such that the weight of each feature source can change dynamically with the real-time fatigue feature measurements. Further, the proposed model can combine the fatigue state at the previous time step in the decision-level fusion to improve the robustness of the fatigue driving recognition. An improved correction strategy of the BPA is also proposed to accommodate the decision conflict caused by external disturbances. Results from field experiments demonstrate that the effectiveness and robustness of the proposed model are better than those of models based on a single fatigue feature and/or single-source information fusion, especially when the most effective fatigue features are used in the proposed model.

## 1. Introduction

Safety is a crucial issue in transportation systems. Fatigue driving is a major cause of road accidents, and can lead to physical injury, death and/or economic loss. The National Highway Traffic Safety Administration (NHTSA) estimates that 100,000 vehicle crashes resulted directly from driver fatigue in the USA, resulting in 1550 deaths, 71,000 injuries, and $12.5 billion in monetary losses annually [1]. Some studies indicate that fatigue driving accounts for 16% of all crashes and over 20% of the crashes on highways [2]. Fatigue driving also increases accident risk four to six times. Therefore, many studies have sought to develop effective and robust fatigue driving recognition models to aid accident risk reduction and enhance driving safety.

Past studies [1,3,4] attempt to recognize fatigue driving from different information sources, such as driver physiological state, facial expression, and vehicle operation condition. Based on the sources of fatigue features, fatigue driving recognition models can be divided into two categories: single-source models and multiple-source models. The single-source models mainly focus on detection and fusion of the fatigue features coming from a single information source. The multiple-source models can fuse multiple fatigue features coming from different information sources.

In the category of single-source models, physiology-based models [3,4,5,6,7,8,9] identify driver’s fatigue states by extracting signals of electroencephalogram (EEG), electrooculogram (EOG), electrocardiogram (ECG), electromyography (EMG) or heart rate variability (HRV). Although these models can accurately recognize fatigue states, electrodes need to contact the skin of the driver for physiological signal detection, which can cause an uncomfortable and annoying feeling. Hence, they are not suitable for fatigue recognition over a long time period.

Facial feature based models [1,10,11,12,13] recognize the fatigue driving by analyzing facial expression changes, such as eye closure duration, blinking, yawning, or eyelid/gaze movement through recognition methods based on computer vision theory. These facial features provide an effective mechanism because the driver’s fatigue state can be measured in a non-intrusive way. However, the robustness of these models may reduce due to environmental interference, such as lighting changes, sudden head movements, and darkness at night.

Another type of single-source models recognizes fatigue driving by detecting vehicle operation condition [14] such as the lane departure degree [15,16] or the variation of steering wheel angle [17,18]. Their accuracy depends on legible lanes and driver driving habits. For example, the applicability of these models may be impeded when a lane marking is blurred or shielded.

As mentioned heretofore, various types of disturbances (such as sudden lighting changes, missing sensor signals, *etc.*) can interfere with fatigue feature measurements. Hence, it is difficult for single-source-based models to accommodate complex environmental changes. To improve the effectiveness and robustness of the fatigue driving recognition under a complex travel environment, models based on multi-source information have been developed. Boyraz *et al.* [19] proposed a recognition model using facial and vehicle behavior features. Yang *et al.* [20] proposed a Dempster-Shafer evidence theory (D-SET) based driver fatigue recognition model by combining contextual features, physiological features, and facial visual features. However, because these models ignore the temporal correlation of driver’s fatigue state, their robustness reduces when some fatigue feature measurements cannot be obtained due to unexpected changes of external travel environment.

Other models attempt to capture the temporal correlation by applying basic probability assignment (BPA) in the information fusion to accommodate the external environment changes. Cheng *et al.* [21] proposed a two-level fusion framework that includes feature-level fusion and decision-level fusion for fatigue driving recognition. However, the accuracy of this fatigue driving recognition model is not reliable because the framework cannot dynamically determine the BPA for each expected fatigue state when multiple fatigue features are fused. Lee *et al.* [22] proposed a fatigue driving recognition model based on dynamic Bayesian network. While the model considers the recognition outcome of the previous time step, the probability weight of each fatigue feature is predetermined according to experts’ subjective experience. The predetermined weights may deteriorate the accuracy of fatigue driving recognition if the experts have not factored the travel environment variation. Yang *et al.* [23] proposed a dynamic Bayesian network model in which the Gaussian distribution function is used to determine probability weights. A first-order hidden Markov model was used to compute the dynamics of the Bayesian network at different time steps. In their model, the probability weights are treated as constants after the assignment. As the probability weight of each fatigue feature in all these models cannot be changed dynamically to adapt to the environmental variation, the static weights may deteriorate the accuracy and robustness of the recognition model.

The literature review heretofore illustrates that the challenges in improving the accuracy and robustness of fatigue driving recognition model arise from disturbances such as sudden lighting changes, missing sensor signals, and incorrect feature measurements. This study seeks to improve the accuracy and robustness of fatigue driving recognition by developing a self-adaptive dynamic recognition model that incorporates the most effective fatigue features. In contrast to existing fatigue driving recognition models, the proposed model introduces a dynamic BPA to the decision-level fusion such that the weight of each feature source can adapt to real-time fatigue feature measurements. Additionally, the proposed model combines the most effective fatigue features in the feature-level fusion and the fatigue state at the previous time step in the decision-level fusion to improve the robustness of fatigue driving recognition. An improved correction strategy of the original BPA is introduced to accommodate the decision conflict caused by external disturbances.

The study contributes to the research on fatigue driving recognition in four aspects. First, the study is the first to determine the dynamic assignment of BPA by carrying out feature-level fusion based on Takagi-Sugeno fuzzy neural network (T-SFNN) [24]. This enables the improvement of robust fatigue driving recognition. Second, in addition to facial expression and vehicle behavior, the fatigue state at the previous time step is also regarded as an evidence source and incorporated into the model, which further enhances the robustness of recognition model through the consideration of the fatigue state correlation in temporal space. Third, a correction strategy for the original BPA is proposed in the decision-level fusion based on D-SET, which resolves the evidence conflict caused by multiple pieces of evidence with different attributes. Fourth, the fatigue driving recognition model is validated against data from field experiments instead of laboratory simulation, which demonstrates the practical applicability of the proposed model.

The remainder of this paper is organized as follows. Section 2 summarizes the fatigue feature measurements that are used to estimate driver fatigue state. Section 3 proposes a self-adaptive dynamic recognition model, including feature-level fusion based on T-SFNN and decision-level fusion based on D-SET. Section 4 demonstrates the effectiveness and robustness of the proposed model using the data collected from field experiments. The final section provides concluding comments.

## 2. Preliminaries

In this study, the fatigue driving state is estimated based on two categories of measurements: driver facial expression and vehicle operation condition. The two categories of measurements are explained in detail in this section.

### 2.1. Facial Feature Based Measurements

Blinking frequency (BF), eye-closed duration (ECD), mean of eye-opened level (MEOL) and yawning frequency (YF) are considered as facial fatigue features in this paper to indicate fatigue state of the driver.

The BF is measured as: $f_{b}=n_{b}/N_{b}$, where *N_b_* is the number of the images captured in a one-minute interval, and *n_b_* is the number of the images where eyes are identified as closed state [25].

The ECD is measured as: $f_{c}=n_{c}/N_{c}$, where *N_c_* is the number of the images captured in a one-minute interval, and *n_c_* is the number of the images where eyes are identified as closed state in a continuous period of two seconds.

The MEOL is defined as: $f_{o}=\sum_{i=1}^{N_{o}}h_{o,i}/N_{o}$, where *h_o,i_* is the height (in pixel) between the upper and lower eyelids in the ith frame image, and *N_o_* denotes the total number of the images captured in a one-minute interval.

The YF is measured as: $f_{m}=n_{m}/N_{m}$, where *n_m_* is the number of images in which yawning is inferred, and *N_m_* denotes the total number of images captured in a one-minute interval. Yawning is inferred by comparing mouth-opening level *r_m_* to threshold *T_m_*. If $r_{m}\geq T_{m}$, then the driver is yawning. The mouth-opening level *r_m_* is defined as: $r_{m}=h_{m}/w_{m}$, where *h_m_* denotes the height between the upper and lower lips, and *w_m_* is the width between the left and right corners of mouth. The values of *h_m_* and *w_m_* can be determined according to literature [26].

### 2.2. Vehicle Behavior Feature Based Measurements

In this study, vehicle behavior features that are used to infer the driver’s fatigue state include percentage of non-steering (PNS), standard deviation of steering-angle (SDSA), frequency of abnormal lane deviation (FALD) and standard deviation of vehicle speed (SDVS).

The PNS is defined as: $p_{a}=n_{a}/N_{a}$, where *N_a_* is the total number of sampled points collected in a ten-second interval, and *n_a_* is the number of points where the angular velocities of steering wheel are within ±0.1 degree/s.

The SDSA is defined as: $s_{a}=\sqrt{\sum_{i=1}^{N_{a}}{\left(x_{a,i}-m_{a}\right)}^{2}/N_{a}}$, where $m_{a}=\sum_{i=1}^{N_{a}}x_{a,i}/N_{a}$, *N_a_* is the number of the samples collected in a ten-second interval, and *x_a,i_* is the angle value of steering wheel of the sample obtained in the *i*th time step.

The FALD is defined as: $f_{l}=n_{l}/N_{l}$, where *N_l_* is the number of images captured in a one-minute interval, and *n_l_* is the number of images where the vehicle is identified as deviating from the lane abnormally. That the vehicle is deviating from lane is judged according to following decision rules [16,27]: $\xi (k)>\lambda_{L}$, or $\xi (k)>\lambda_{R}$, where, $\xi (k)=(\frac{\pi }{2}-{\text{θ}}_{L}(k))/({\text{θ}}_{R}(k)-\frac{\pi }{2})$, θ*_L_*(*k*) and θ*_R_*(*k*) represent the slope angle of left and right lane lines in the *k*th frame of image, respectively. Parameters *λ_L_* and *λ_R_* are the thresholds of left deviation and right deviation, respectively.

The SDVS is defined as: $v_{s}=\sqrt{\frac{1}{n}\sum_{i=1}^{n}{\left(v_{v,i}-m_{v}\right)}^{2}}$, where ν_s_ represents the standard deviation of vehicle speeds, $m_{v}=\sum_{i=1}^{N_{v}}v_{v,i}/N_{v}$ represents the average speed during a ten-second interval, *N_ν_* is the number of sample points in a ten-second interval and *ν_ν,i_* is the speed value gained by global positioning system (GPS) at time *i*.

The measurements defined in this section will be used to determine fatigue features of the self-adaptive dynamic recognition model, which will be incorporated into the proposed recognition model for carrying out real-time fatigue driving recognition as input parameters.

## 3. Self-Adaptive Dynamic Fatigue Recognition Model

Because the multiple fatigue features determined in Section 2 come from two different fatigue feature sources, they can comprehensively reflect driver’s fatigue state. In the following recognition model, we will incorporate all the measured fatigue features to enhance the reliability and robustness of fatigue driving recognition. However, if multiple fatigue features from different information sources are incorporated into a single-level recognition model, it can result in a complex model structure and weak system stability. To enhance the model performance, we propose a self-adaptive dynamic recognition model with two levels of fusion, which includes the feature-level fusion based on T-SFNN and decision-level fusion based on D-SET. In the feature-level fusion, T-SFNN is used to fuse multiple fatigue features obtained from facial expression and vehicle operation behavior to provide a dynamic BPA for the decision-level fusion. In the decision-level fusion, D-SET is used to fuse three pieces of evidence derived from three different information sources to improve the robustness of fatigue driving recognition.

### 3.1. General Recognition Framework

**Figure 1:**
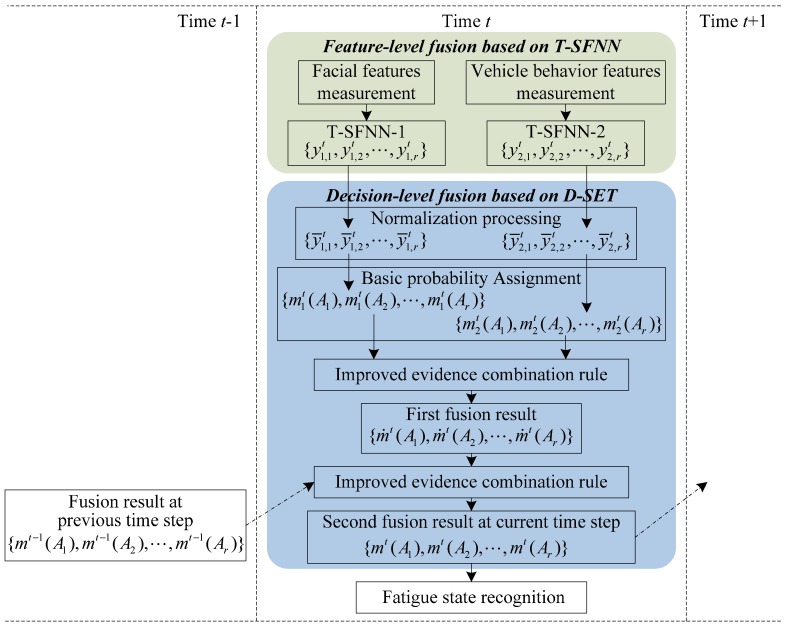
Framework of the fatigue recognition model based on multi-source information and two levels of fusion.

The model structure of the proposed self-adaptive dynamic recognition model is summarized in Figure 1. The key components and recognition procedures of the fatigue driving recognition model are as follows.

(i) Feature-level fusion based on T-SFNN: First, the most effective fatigue features are measured in real-time, and provide two types of information: driver’s facial expression and vehicle behavior. Second, facial features and vehicle behavior features are inputs to two T-SFNN models: T-SFNN-1 and T-SFNN-2, respectively. The outputs of T-SFNN-1 and T-SFNN-2 at time *t* are considered as the inputs to the decision-level fusion based on D-SET, which can realize dynamic BPA assignments in the proposed model as shown in Figure 1.
(ii) Decision-level fusion based on D-SET: First, the outputs of T-SFNN-1 and T-SFNN-2 are normalized. Second, as shown in Figure 1, the normalized results are regarded as two pieces of evidence for decision-level fusion. Third, the two pieces of evidence are fused by an improved evidence combination rule. The first fusion result is regarded as an intermediate result which is used as input to the second fusion. Fourth, the first fusion result is fused with the fusion result at the previous time step *t −* 1. The second fusion result is regarded as the final decision-level fusion result at time step *t*. Fifth, the decision-level fusion result at time step *t* is recorded to be used as a piece of evidence in the decision-level fusion at next time step *t + 1*, as illustrated in Figure 1.
(iii) Output recognition result: The driver’s fatigue state at time step *t* is determined based on the decision-level fusion result and the fatigue decision rule.

In the following sections, we will discuss the feature-level fusion and decision-level fusion in detail.

### 3.2. Feature-Level Fusion

The T-SFNN combines the advantages of fuzzy logic (in processing vague and uncertain information) and neural networks (in providing good learning capabilities) [24]. Compared with the conventional approaches, e.g., fuzzy logic, it has been shown that TSFNN can achieve better performance in mathematical function approximation in modeling highly nonlinear systems [24]. Therefore, the feature-level fusion based on T-SFNN model can generate more accurate BPA for decision-level fusion. In this study, T-SFNN-1 and T-SFNN-2 models are used to generate real-time BPAs by fusing facial and vehicle behavior features, respectively. For simplifying the model structure, in each T-SFNN model, the subtractive clustering algorithm (SCA) is used to obtain the optimal T-SFNN structure. The improved particle swarm optimization (IPSO) algorithm is also employed to train the T-SFNN for acquiring accurate network parameters. Therefore, based on the simplified structure and optimized parameters, the two T-SFNN models can enable more accurate and reliable BPAs.

#### 3.2.1. Structure of T-SFNN

**Figure 2:**
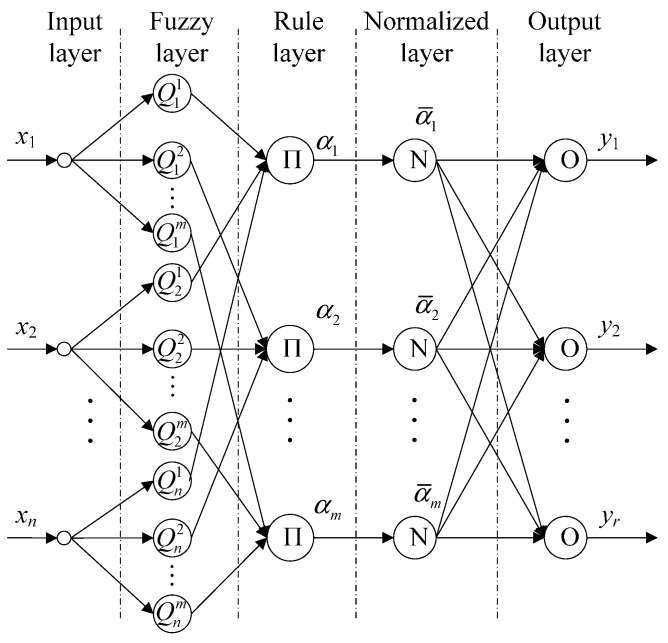
Structure of T-SFNN.

Traditional T-SFNN consists of antecedent network and consequent network with complex network structure and numerous network parameters to be determined [24]. To simplify the network structure and improve computational efficiency, traditional T-SFNN is improved by reorganizing the network structure. As shown in Figure 2, the improved T-SFNN structure is composed of five layers and the function of each layer is as follows:

The first layer is the input layer, where each node represents an input variable.

The second layer is the fuzzy layer, where each node represents one linguistic value of each input variable. Each node expresses a fuzzy subset $Q_{i}^{j}$ described by Gaussian membership function: $\mu_{i}^{j}=\mu_{Q_{i}^{j}}(x_{i})=\exp[-{\left(x_{i}-c_{ij}\right)}^{2}/\sigma_{ij}^{2}]$, i=1,2,…,n, j=1,2,…,m, where *m* indicates the number of linguistic values of each input variable, *n* is the number of nodes in the input layer, and *c_ij_* and *σ_ij_* are defined as the center and width of the Gaussian membership function, respectively.

The third layer is the rule layer which is used to calculate the firing strength α_*j*_ of every fuzzy rule. Each node in the layer represents one fuzzy rule, ${\text{α}}_{j}=\prod_{i}\mu_{i}^{j}$, j=1,2,…,m.

The fourth layer is the normalized layer which is used to calculate the normalized firing strength ${\bar{\text{α}}}_{j}$ of the corresponding rule: ${\bar{\text{α}}}_{j}={\text{α}}_{j}/\sum_{i=1}^{m}{\text{α}}_{i}$, j=1,2,…,m.

The fifth layer is the output layer which is used to provide the reference result of the whole system. Each node in this layer represents one output variable, $y_{k}=\sum_{j=1}^{m}w_{kj}{\bar{\text{α}}}_{j}$, where *w_kj_* is defined as the weight of neural network, k=1,2,⋯,r, where *r* is the number of output variables.

#### 3.2.2. Learning Algorithm

For the T-SFNN shown in Figure 2, when the number of input variables and fuzzy sets for each input becomes more than four or five, it will result in the problem of combinatorial explosion of rules, namely the so-called curse of dimensionality. In order to optimize the inner structure of the T-SFNN and obtain optimum network parameters, we introduce a structure learning algorithm based on SCA and parameter learning algorithm based on IPSO.

For the structure learning, the SCA is used to optimize the network structure according the cluster amount extracted from the given training samples [28]. By clustering, let the number of linguistic values of each input node and the number of fuzzy rules be equal to the number of the extracted clusters, which will significantly reduce the number of parameters of the TSFNN. The algorithm used in this study can be succinctly described as follows. Suppose that $C_{j}=\{x_{1,j},x_{2,j},\cdots ,x_{n,j}\}$, $j=1,2,\cdots ,c_{R}$ is the *j*th cluster center obtained by SCA, where *c_R_* is the number of clusters determined by the SCA. Assuming that *C_1_* is the first cluster center obtained, and *C_s_* is the nearest cluster center to *C_1_*, *i.e.*, the Euclidean distance between *C_1_* and *C_S_* is the shortest, where, $C_{1}=\{x_{1,1},x_{2,1},\cdots ,x_{n,1}\}$, $C_{S}=\{x_{1,S},x_{2,S},\cdots ,x_{n,S}\}$, $s=2,\cdots ,c_{R}$. Accordingly, by letting $m=c_{R}$, ${c^{\prime }}_{ij}$ = $x_{i,j}$ and ${\sigma^{\prime }}_{ij}$ = $\left|x_{1,j}-x_{S,j}\right|/2$, the initial value ${c^{\prime }}_{ij}$ and ${\sigma^{\prime }}_{ij}$ of parameters *c_ij_* and *σ_ij_* are determined.

For the parameter learning, the back propagation (BP) algorithm [29] and genetic algorithm (GA) [30] have been proven to have superior performance for specific problems. However, because of gradient descent, the BP method has been criticized for its shortcomings of becoming stuck in local minima and sensitivity to the initial values. The main drawbacks of GA are its huge computation time and slow convergence near the optimum. When compared to BP and GA, the IPSO is simpler in operation and easier to understand owing to a smaller number of free tunable parameters. Therefore, we introduce the IPSO algorithm to determine network parameters and network weights of T-SFNN. First, determine the initial value of network parameters. The network parameters are initialized as ${c^{\prime }}_{ij}$ and ${\sigma^{\prime }}_{ij}$ according to the SCA, and network weight *wkj* is initialized as a random value ${w^{\prime }}_{kj}$ in the range of [0,1]. Second, the IPSO algorithm is used to obtain the optimal solutions of network parameters and network weight. In the IPSO, according to the fitness function shown in Equation (1), the optimal solutions of ${\hat{c}}_{ij}$, ${\hat{\sigma }}_{ij}$ and ${\hat{w}}_{kj}$ are obtained [28].

(1) $$f(x)=\frac{1}{N}\sum_{k=1}^{N}\sum_{j=1}^{r}{\left(y_{k,j}-{\hat{y}}_{k,j}\right)}^{2}$$

where *N* is the number of training samples, *r* is the number of the nodes in the output layer, and *y_k,j_* and ${\hat{y}}_{k,j}$ are the actual and desired outputs, respectively.

### 3.3. Decision-Level Fusion

Multi-source information fusion based on Bayesian networks has been applied to the field of fault diagnosis and achieved good diagnosis outcomes [31]. Compared to other statistical inference methods, D-SET is closer to human perception and reasoning process, and can fuse information collected from different sources to infer results with some degree of certainty. Unlike the Bayesian reasoning model that depends on the prior probability, D-SET is more suitable for practical applications [32]. Therefore, based on the D-SET, we propose an improved decision-level fusion method to combine three pieces of evidence from three different information sources. The proposed decision-level fusion method includes three steps: (i) dynamic BPA calculation; (ii) combination of evidence; and (iii) fatigue state decision.

#### 3.3.1. Dynamic BPA Calculation

The key role of D-SET is to model the knowledge of the problem by initializing the BPA based on the evidence provided by different sensors. Assume *m*(*A_i_*) is the BPA of the ith hypothesis *A_i_* in the frame of discernment Θ of D-SET, where Θ is a finite non-empty set of mutually exclusive alternatives containing every possible hypothesis *A_i_* [32]. Notation m is a mass function, *m*(*·*): $2^{\text{Θ}}\to [0,1]$, which satisfies: $m(\text{∅})=0,\sum_{A_{i}\subseteq \text{Θ}}m(A_{i})=1$ [33]. In this paper, *A_i_* represents the evaluated fatigue state according to a certain fatigue assessment method.

Generally, the BPA is determined by experts based on their experience. However, such a subjective and static BPA assignment will reduce robustness of the proposed model in practice. To overcome the difficulty, the BPA *m*(*A_i_*) for each evidence source is dynamically assigned by the T-SFNN model according to its real-time output results in this study. The *m*(*A_i_*) can be calculated by:

(2) $$m(A_{i})=y_{i}/\sum_{i=1}^{r}y_{i},\;i=1,2,\cdots ,r$$

where *y_i_* is the output of the ith node component in output layer of the T-SFNN.

#### 3.3.2. The Improved Evidence Combination

Denote Θ = {$A_{1}$, $A_{2}$, ⋯, $A_{r}$} as fatigue state set, e = {$e_{1}$, $e_{2}$, ⋯, $e_{t}$} as evidence set, and *m_1_*(*·*), *m_2_*(*·*), ⋯, *m_t_*(*·*) as the BPA mass functions over Θ. The Dempster combination rule for any two pieces of evidence in the evidence set *e* is [34,35]:

(3) $$\begin{array}{lll} m(A_{k}) & =m_{1}(A_{k})⊕m_{2}(A_{k}) & \\ & =\left\{ \begin{array}{ll} \frac{1}{1-K}\sum_{A_{i}\cap A_{j}=A_{k}}m_{1}(A_{i})m_{2}(A_{j}), & \forall A_{k}\in \text{Θ},\\ 0, & A_{k}\notin \text{Θ} \end{array}\right. \end{array}$$

where $K=\sum_{A_{i}\cap A_{j}=\text{∅}}m_{1}(A_{i})m_{2}(A_{j})$, which denotes the conflict degree between two pieces of evidence.

In Equation (3), *K* = 1implies that the two pieces of evidence are completely conflicting. Under this situation, the aforementioned combination rule becomes invalid. If K→1, then two pieces of evidence have high conflict and an illogical result may be produced by this rule [36]. Therefore, we should identify whether a conflict happens before conducting evidence combination based on the conflict degree *K*. The improved evidence combination rule for any two pieces of evidence is given: if $0<K<k_{T}$, then the original BPA is available in the following evidence combination, where *kT* is a predetermined threshold. Otherwise, if $k_{T}\leq K<1$, then the original BPA needs to be modified by introducing a belief factor. The modified BPA is updated as:

(4) $$\left\{ \begin{array}{ll} {m^{\prime }}_{i}(A_{j})=m_{i}(A_{j}){\text{η}}_{i}, & A_{j}\in \text{Θ}\\ {m^{\prime }}_{i}(\text{Θ})=1-\sum_{j=1}^{N}m_{i}(A_{j}){\text{η}}_{i}, & A_{j}\notin \text{Θ} \end{array}\right.$$

In Equation (4), the belief factor *η_i_* can be obtained through the following steps:

Step 1: Calculate the Euclidean distance *d_i,j_* between any two pieces of evidence *e_i_* and *e_j_*: $d_{i,j}=\sqrt{\frac{1}{2}({\left\|m_{i}\right\|}^{2}+{\left\|m_{j}\right\|}^{2}-2\langle m_{i},m_{j}\rangle )}$, where ${\left\|m_{i}\right\|}^{2}$ = $\langle m_{i},m_{i}\rangle$, ${\left\|m_{j}\right\|}^{2}$ = $\langle m_{j},m_{j}\rangle$, and $\langle m_{i},m_{j}\rangle$ represents the dot product between two vectors *m_i_* and *m_j_*.

Step 2: Define confidence level $c_{i,j}=\sqrt{K_{i,j}\cdot d_{i,j}}$, where $K_{i,j}=\sum_{A_{i}\cap A_{j}=\text{∅}}m_{i}(A_{i})m_{j}(A_{j})$.

Step 3: The average confidence level *c_i_* can be defined as: $c_{i}=\frac{1}{n}\sum_{j=1}^{n}c_{i,j}$.

Step 4: The belief factor *η_i_* is determined by:

(5) $${\text{η}}_{i}=(1-c_{i})e^{c_{i}}$$

#### 3.3.3. Fatigue State Decision

After conducting the evidence combination, we can determine the driver’s fatigue state based on the following decision rule:

(6) $$\left\{ \begin{array}{l} m(A_{F})-m(A_{S})>\varepsilon_{T1}\\ m(A_{F})>\varepsilon_{T2} \end{array}\right.$$

where *m*(*A_F_*) is the largest probability value, $m(A_{F})=\max\{m(A_{k}),A_{k}\in \text{Θ}\}$, *m*(*A_S_*) is the second largest probability value, $m(A_{S})=\max\{m(A_{k}),A_{k}\in \text{Θ},and\;A_{k}\neq A_{F}\}$, *ε_T_*_1_ and *ε_T_*_2_ are the given thresholds in advance, k=1,2,⋯,r.

If *A_F_* satisfies the decision rule Equation (6), then it is regarded as the driver’s fatigue state; otherwise, the fatigue state recognized in the previous time step is outputted as the current fatigue state.

## 4. Experiment Results and Analysis

This section presents examples based on field experiments to demonstrate the effectiveness and robustness of the proposed fatigue recognition model by applying the field experiment data to the proposed model and comparing its performance with those of other models.

### 4.1. Experiment Design

**Figure 3:**
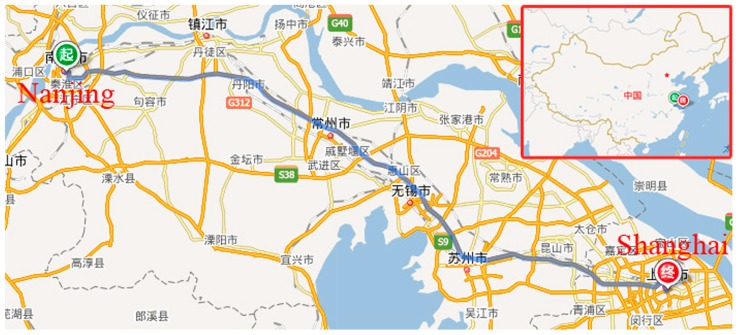
Experiment route.

The experiments were carried out on the Nanjing-Shanghai expressway (China), which is highlighted in blue in Figure 3. Three men and two women with ages ranging from 25 to 32 and more than three years of driving experience participated in the experiments. Alcohol, tea, coffee, drugs or other drinks that can cause excitement to the nervous system were prohibited for 24 h prior to the experiments. The experiments were performed after informed consent on the procedures of the experiments was received from all participants.

The experiments were carried out from 12:00 p.m. to 3:00 p.m. on 25 November 2014 for all participants, because previous studies show that drivers can become easily fatigued during this period of the day [37]. To avoid traffic accidents, the experiments were conducted on road sections with few vehicles. In addition, an experienced driver was asked to sit in the front passenger seat to warn the participant or execute necessary emergency maneuvering.

### 4.2. Fatigue State Assessment

The effective classification of a driver’s fatigue state during driving is a difficult problem. The existing studies focus on subjective assessments on fatigue driving by observing some phenomena, such as a driver’s facial expression, operational behavior and self-evaluation of fatigue, which may result in an inaccurate classification. In view of objectivity of the EEG detection, we propose a comprehensive assessment method based on objective detection and subjective assessment to classify fatigue states, which will improve the accuracy of fatigue state classification. The main steps of the comprehensive assessment method are as follows:

(i) Observer Assessment: Fatigue states of participants are evaluated by three observers according to the video of facial expression and driving behavior captured by three cameras. Of the three cameras, one is placed towards the driver’s face, another towards the steering-wheel, brake pedal, and gearshift, and the third one towards the front lane. Every video is evaluated by the three observers according to the fatigue state characteristics described in Table 1. If the fatigue state of the participant is evaluated as “Non-Fatigue (NF)”, then let *s_i,j_* = 0, where *s_i,j_* indicates the score of the *j*th video evaluated by the ith observer; if the fatigue state is determined as “Moderate Fatigue (MF)” or “Severe Fatigue (SF)”, then let *s_i,j_* = 1 or *s_i,j_* = 2, respectively. To reduce subjectivity of assessment, the average of the scores given by three observers is regarded as the final score, *i.e.*, ${s^{\prime }}_{j}=\text{INT}\left\lfloor (\sum_{i=1}^{3}s_{i})/3\right\rfloor$, where, INT⌊⋅⌋ is a rounding operator. The fatigue state of the participant in the *j*th video is evaluated according to the average ${s^{\prime }}_{j}$. If ${s^{\prime }}_{j}=0$, then the fatigue state is “NF”; if ${s^{\prime }}_{j}=1$, then fatigue state is “MF”; if ${s^{\prime }}_{j}=2$, then fatigue state is “SF”.

**Table 1:** Video observation based fatigue assessment.

Fatigue State	State Description	Score
NF	Eyes are active and concentrated; sits straight, operation of hands and feet is agile, keeps focusing on the front, and stable vehicle speeds.	1
MF	Eyes, mouth and hands move slightly unconsciously, yawns, head swings, adjusts the sitting position discontinuously, consistent operation of hands and feet; eye movement declines, eyelids sometimes close, frequently yawns, operations of hands and feet are not agile, not too stable vehicle speeds.	2
SF	Eyelids always closed, eyes are dull, nods, winks and shakes the head to resist fatigue, uncoordinated operation of hands and feet; eyes suddenly open after closing for a period, head droop and body incline begin to occur, hands and feet operate unconsciously, unstable speeds and zigzag routing occur.	3

(ii) EEG Assessment: The objective assessment based on EEG is conducted to evaluate the fatigue state of the participants. The value of *r_α,θ,β_* is considered as an index to reflect the fatigue state, which is defined as [38]:
  (7) $$r_{\text{α},\text{θ},\text{β}}=\frac{P_{\text{α}}+P_{\text{θ}}}{P_{\text{β}}}$$
  where, *P_α_*, *P_θ_* and *P_β_* are the power spectra of the three wave bands of α, θ and β, respectively, and the frequency ranges of α, θ and β bands are (4–8 Hz), (8–13 Hz), and (13–22 Hz), respectively. The fatigue state is categorized into three levels according to the value of *r_α,θ,β_* defined in Table 2.
(iii) Self-Assessment: Let the participant make a self-assessment of fatigue state according to his/her current physical, physiological and psychological situations. Based on the scores gained and the 7-point Stanford Sleepiness Scale (SSS) table [39], fatigue is then rated into one of three states: “NF” (1–2 points), “MF” (3–5 points), and “SF” (6–7 points).

**Table 2:** EEG detection based fatigue assessment.

Fatigue State	${\text{r}}_{\text{α,θ,β}}$
NF	$r_{\text{α},\text{θ},\text{β}}<3$
MF	$3\leq r_{\text{α},\text{θ},\text{β}}<4$
SF	$r_{\text{α},\text{θ},\text{β}}\geq 4$

(iv) Comprehensive Assessment: The participant’s fatigue state is determined according to the results from steps (i)–(iii). If the fatigue state results from all three methods are consistent, then the assessment result obtained is considered valid and correct, which will be regarded as the actual fatigue state of the participant. Otherwise, it is removed from the ground truth set.

To ensure the reliability of the assessment, the assessments based on observer and EEG detection must be implemented simultaneously, and the self-assessments based on SSS table need to be carried out within one minute after the other two assessments are accomplished.

The proposed assessment method will be used to select the most effective fatigue features and provide ground truth data for following model calibration and verification.

### 4.3. Data Collection

**Figure 4:**
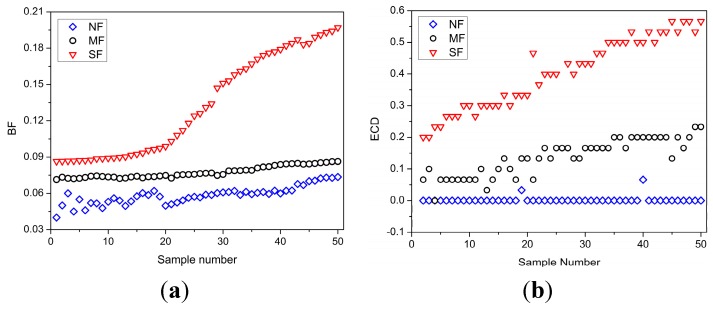

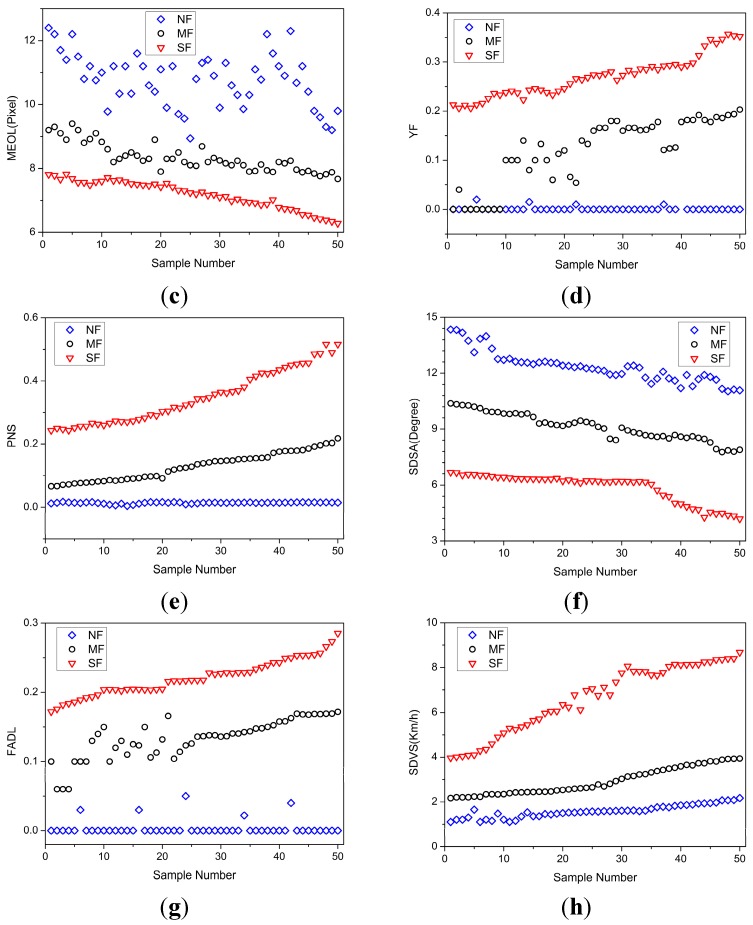
Fatigue feature measurement. (**a**) F measurement; (**b**) ECD measurement; (**c**) MEOL measurement; (**d**) YF measurement; (**e**) PNS measurement; (**f**) SDSA measurement; (**g**) FADL measurement; (**h**) SDVS measurement.

According to the detection methods described in Section 2, eight fatigue features were measured. In total 1200 data points were collected, each of which includes the eight fatigue features. These data were separated into two sets: (i) the training set that included 800 data points for model calibration; and (ii) the remaining 400 data points for model verification. Further, based on the comprehensive assessment method proposed in Section 4.2, the measurement results of every fatigue feature were divided into three groups according to their corresponding fatigue states, “NF”, “MF” and “SF”. We select 150 data points randomly from the 1200 data points illustrated in Figure 4. For each fatigue feature, 50 data points each belong to the “NF”, “MF” and “SF” groups.

As seen from the Figure 4, the fatigue states cannot be identified easily according to the measurement values of fatigue features. For example, theoretically, the data in the “NF” state should have larger values than the data in the “MF” state, and the data in the “MF” state should have larger values than the data in the “SF” state in MEOL measurement shown in Figure 4c. However, some data points in the “NF” state have smaller values than the data in the “MF” state. Therefore, in order to recognize the driver’s fatigue state more effectively, the effectiveness of these measured fatigue features needs to be analyzed further.

### 4.4. Fatigue Feature Identification

As previously mentioned, not all fatigue features measured in the previous section can be used to reliably reflect fatigue state of driver. In addition, too many fatigue features are likely to cause data redundancy and result in heavy computational burden, which may preclude real-time recognition. Therefore, the fatigue features presented in the previous section need to be analyzed further to eliminate irrelevant fatigue features.

To select the most effective fatigue features, the correlation between fatigue features and fatigue driving need to be identified. Pearson test is regarded as an effective method to carry out the correlation analysis. The associated procedure is as follows:

(i) The Kolmogorov-Smirnov test [40]: It is used to estimate whether every fatigue feature follows a normal distribution. For the features not normally distributed, they will be normalized through logarithmic transformation.
(ii) Pearson test [40]: It is used to verify the correlation between a fatigue feature and fatigue.
(iii) Feature selection: The most effective fatigue features are selected according to the correlation calculated by Pearson test. If the statistic value of a fatigue feature is smaller than the quantile value, it is considered uncorrelated to fatigue driving and removed from the candidate set of fatigue features.

Table 3 shows the verification results based on the Kolmogorov-Smirnov test. It shows that the statistics of BF, ECD, MEOL, YF, PNS, SDSA, FALD and SDVS are smaller than the quantile value, implying that they are all normally distributed.

**Table 3:** Normal distribution testing of fatigue features.

Fatigue Feature Parameters	Kolmogorov-Smirnov Testing				
	Mean	Standard Deviation	Statistic Value	Significance Level	Statistic Quantile Value
BF	0.1237	0.0214	0.0576	0.05	0.1297
ECD	0.3178	0.0414	0.0742	0.05	0.1297
MEOL	10.194	2.347	0.069	0.05	0.1297
YF	0.2074	0.0213	0.0703	0.05	0.1297
PNS	0.2857	0.0278	0.0583	0.05	0.1297
SDSA	12.69	2.4157	0.0623	0.05	0.1297
FALD	0.6138	0.0872	0.0718	0.05	0.1297
SDVS	7.315	1.0773	0.0715	0.05	0.1297

The correlation coefficient between each fatigue feature and fatigue state is computed by using the Pearson test and the associated statistics are summarized in Table 4. Table 4 illustrates that the correlation coefficients of MEOL and SDSA are negative and the other six fatigue features are positively correlated to fatigue. Further, the absolute value of the correlation coefficient of MEOL is very small and its statistic value is less than the corresponding statistic quantile value. It indicates that MEOL is not significantly correlated with fatigue. Therefore, the seven fatigue features that have significant correlation with fatigue are selected as the most effective fatigue features, and the fatigue feature MEOL is excluded.

**Table 4:** Correlation analysis between fatigue features and fatigue.

Fatigue Features	Fatigue			
	Correlation Coefficient	Significance Level	Statistic Value	Statistic Quantile Value
BF	0.787	0.05	6.362	1.982
ECD	0.389	0.05	3.137	1.982
MEOL	−0.034	0.05	1.107	1.982
YF	0.613	0.05	4.814	1.982
PNS	0.713	0.05	6.324	1.982
SDSA	−0.622	0.05	4.896	1.982
FALD	0.562	0.05	4.528	1.982
SDVS	0.675	0.05	5.968	1.982

### 4.5. Feature-Level Fusion Results

There are four main steps in the feature-level fusion. First, according to the number of the effective fatigue features and the fatigue state of sample data, a 3-input and 3-output neural network of T-SFNN-1 is determined, where input variables *x_i,_*_1_ (*i* = 1, 2, 3) are used to represent the BF, ECD and YF measurements, respectively. A 4-input and 3-output neural network of T-SFNN-2 is determined, where input variables *x_i,_*_2_ (*i* = 1, 2, 3, 4) are used to represent the PNS, SDSA, FALD and SDVS measurements, respectively. Output variables *y_j_* (j = 1, 2, 3) represent the probabilities of the three fatigue states, *i.e.*, the “NF”, “MF”, and “SF”, respectively. The probabilities are assigned according to the fatigue state of the sample data in training the T-SFNN. If the data point in the training set is evaluated as a certain fatigue state by the proposed comprehensive assessment method, then the output variable related to the evaluated fatigue state is assigned a larger probability value. The remaining probability value is assigned equally to the two output variables related to the other two fatigue states. For example, if the fatigue state of the ith sample data is evaluated as “NF”, then we can let *y*_1_(*i*) = 0.8, *y*_2_(*i*) = 0.1, *y*_3_(*i*) = 0.1; if the fatigue state is “MF”, then let *y*_1_(*i*) = 0.1, *y*_2_(*i*) = 0.8, *y*_3_(*i*) = 0.1; if the fatigue state is “SF”, then let *y*_1_(*i*) = 0.1, *y*_2_(*i*) = 0.1, *y*_3_(*i*) = 0.8.

Second, based on the SCA and sample data, determine the structures of T-SFNN-1 and T-SFNN-2. 480 sample data points are selected from the training set and are used to carry out the SCA. Based on the SCA, these data points for T-SFNN-1 and T-SFNN-2 are divided into 3 clusters. Therefore, the number of fuzzy rules is determined as 3 and the number of the linguistic values for every input variable is also 3 in both T-SFNN-1 and T-SFNN-2. The network structures of the T-SFNN-1 and the T-SFNN-2 without and with the SCA are shown in Table 5.

Table 5 illustrates that the structures of T-SFNN-1 and T-SFNN-2 have been improved when the SCA is adopted. The total number of parameters to be determined in the training stage decreases significantly.

**Table 5:** Comparison of network structure of T-SFNN without and with SCA.

Parameters of T-SFNN	T-SFNN-1		T-SFNN-2	
	Without SCA	With SCA	Without SCA	With SCA
Input-output space	3 inputs, 3 output	3 inputs, 3 output	4 inputs, 3 output	4 inputs, 3 output
Shape of membership function	Gaussian	Gaussian	Gaussian	Gaussian
Number of linguistic values	3	3	3	3
Number of fuzzy rules	27	3	81	3
Number of parameters for training	99	27	267	33

**Figure 5:**
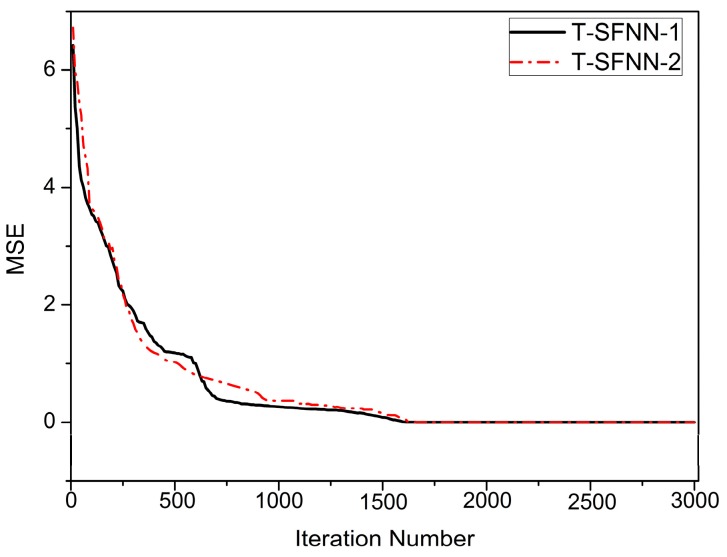
MSE curves based on IPSO for T-SFNN-1 and T-SFNN-2.

Third, T-SFNN-1 and T-SFNN-2 are trained to obtain the optimal network parameters using the IPSO algorithm based on 360 training sample data points, including 120 “NF”, 120 “MF” and 120 “SF” samples, respectively. The convergence curves measured by the mean square error (MSE) are shown in Figure 5. As illustrated by Figure 5, the MSE values of T-SFFN-1 and T-SFNN-2 decline to 10^−4^ after 1642 iterations based on the IPSO algorithm. Therefore, the network parameters can be efficiently determined using the IPSO algorithm.

Fourth, output the result of feature-level fusion. To verify the proposed model, another 200 sample data points selected from the model verification set are used to compute the output results of T-SFNN-1 and T-SFNN-2. For illustration purposes, 6 of the 200 output results are shown in Table 6, where $\left\{y_{1,1}^{t},y_{1,2}^{t},y_{1,3}^{t}\right\}$ and $\left\{y_{2,1}^{t},y_{2,2}^{t},y_{2,3}^{t}\right\}$ represent the output results of T-SFNN-1 and T-SFNN-2 at time *t*, respectively.

**Table 6:** Feature-level fusion results.

Index	$\text{\{}y_{\text{1,1}}^{t}\text{,}y_{\text{1,2}}^{t}\text{,}y_{\text{1,3}}^{t}\text{\}}$	$\text{\{}y_{\text{2,1}}^{t}\text{,}y_{\text{2,2}}^{t}\text{,}y_{\text{2,3}}^{t}\text{\}}$
1	{0.812, 0.087, 0.103}	{0.782, 0.311, 0.074}
2	{0.203, 0.763, 0.052}	{0.402, 0.432, 0.207}
3	{0.237, 0.624, 0.178}	{0.383, 0.552, 0.134}
4	{0.412, 0.488, 0.106}	{0.721, 0.234, 0.071}
5	{0.127, 0.073, 0.811}	{0.442, 0.551, 0.107}
6	{0.292, 0.457, 0.393}	{0.112, 0.476, 0.389}

### 4.6. Decision-Level Fusion Results

The normalized results of $\left\{{\bar{y}}_{1,1}^{t},{\bar{y}}_{1,2}^{t},{\bar{y}}_{1,3}^{t}\right\}$ and $\left\{{\bar{y}}_{2,1}^{t},{\bar{y}}_{2,2}^{t},{\bar{y}}_{2,3}^{t}\right\}$ at time *t* are used as two pieces of evidence {*m^t^*(*A_1_*), *m^t^*(*A_2_*), *m^t^*(*A_3_*)} $\left\{m_{1}^{t}(A_{1}),m_{1}^{t}(A_{2}),m_{1}^{t}(A_{3})\right\}$, $\left\{m_{2}^{t}(A_{1}),m_{2}^{t}(A_{2}),m_{2}^{t}(A_{3})\right\}$) in the first evidence fusion, *i.e.*, let $m_{1}^{t}(A_{1})$ = ${\bar{y}}_{1,1}^{t}$, $m_{1}^{t}(A_{2})$ = ${\bar{y}}_{1,2}^{t}$, $m_{1}^{t}(A_{3})$ = ${\bar{y}}_{1,3}^{t}$. The first evidence fusion result {*ṁ^t^*(*A_1_*), *ṁ^t^*(*A_2_*), *ṁ^t^*(*A_3_*)} is obtained by carrying out the Dempster combination rule shown in Equation (3). Similarly, for the {*ṁ^t^*(*A_1_*), *ṁ^t^*(*A_2_*), *ṁ^t^*(*A_3_*)} and the recognition result {*m^t-1^*(*A_1_*), *m^t-1^*(*A_2_*), *m^t-1^*(*A_3_*)} at time step *t − 1*, the same computations are conducted. The decision-level fusion result {*m^t^*(*A_1_*), *m^t^*(*A_2_*), *m^t^*(*A_3_*)} at time *t* is obtained by implementing the Dempster combination rule in Equation (3) based on the normalized fusion results of $\left\{{\bar{\dot{m}}}^{t}(A_{1}),{\bar{\dot{m}}}^{t}(A_{2}),{\bar{\dot{m}}}^{t}(A_{3})\right\}$ and $\left\{{\bar{m}}^{t-1}(A_{1}),{\bar{m}}^{t-1}(A_{2}),{\bar{m}}^{t-1}(A_{3})\right\}$. Finally, we can infer the fatigue state *S_F_* of the driver from the decision-level fusion result at the current time step according to Equation (6).

The performance of the proposed model is verified by comparing the fatigue state inferred by the decision-level fusion with that evaluated by the comprehensive assessment method. For the selected 200 data points, 192 are correctly recognized by the proposed model, which demonstrates its effectiveness.

Further, the selected 200 data points were used to verify the robustness of the proposed model under disturbance. Table 7 summarizes the recognition results of the first evidence fusion based on the results shown in Table 6, where ${s^{\prime }}_{F}$ represents the driver’s fatigue state deduced from {*ṁ^t^*(*A_1_*), *ṁ^t^*(*A_2_*), *ṁ^t^*(*A_3_*)} according to the proposed decision rule in Equation (6), *S_EEG_* represents the fatigue state evaluated by the proposed comprehensive assessment method, and $K^{\prime }$ represents the conflict degree between two pieces of evidence in the first evidence fusion. Table 8 shows the recognition results of decision-level fusion of the 6 examples, where *K* represents the conflict degree calculated in the decision-level fusion.

**Table 7:** Recognition results of the first evidence fusion.

Index	$\text{\{}{\bar{y}}_{\text{1,1}}^{t}\text{,}{\bar{y}}_{\text{1,2}}^{t}\text{,}{\bar{y}}_{\text{1,3}}^{t}\text{\}}$	$\text{\{}{\bar{y}}_{\text{2,1}}^{t}\text{,}{\bar{y}}_{\text{2,2}}^{t}\text{,}{\bar{y}}_{\text{2,3}}^{t}\text{\}}$	$K^{\prime }$	{*ṁ^t^*(*A_1_*), *ṁ^t^*(*A_2_*), *ṁ^t^*(*A_3_*)}	${s^{\prime }}_{F}$	*s_EEG_*
1	{0.810, 0.087, 0.103}	{0.670, 0.266, 0.064}	0.441	{0.971, 0.476, 0.012}	NF	NF
2	{0.199, 0.750, 0.051}	{0.386, 0.415, 0.199}	0.603	{0.193, 0.784, 0.026}	MF	MF
3	{0.228, 0.601, 0.171}	{0.359, 0.516, 0.125}	0.587	{0.198, 0.751, 0.052}	MF	MF
4	{0.410, 0.485, 0.105}	{0.703, 0.228, 0.069}	0.593	{0.708, 0.272, 0.018}	NF	NF
5	{0.126, 0.072, 0.802}	{0.402, 0.501, 0.097}	0.835	{0.307, 0.219, 0.471}	SF	SF
6	{0.256, 0.400, 0.344}	{0.115, 0.487, 0.398}	0.640	{0.082, 0.541, 0.380}	MF	SF

As shown by Table 7, the first evidence fusion results differentiate the fatigue states more clearly than the results provided by the feature-level fusion shown in Table 6. For example, for the 2nd sample data point in Table 6, the output value $y_{1,2}^{t}$ (0.763) of T-SFNN-1 is larger than the $y_{1,1}^{t}$ (0.203) and $y_{1,3}^{t}$ (0.052); that the fatigue state belongs to “MF” can be recognized easily. By contrast, the difference between $y_{2,1}^{t}$ (0.402) and $y_{2,2}^{t}$ (0.432) is small. Hence, it is difficult to conclude the driver’s fatigue state based on T-SFNN-2. Nevertheless, after the first evidence fusion is conducted as shown in Table 7, the probability *ṁ^t^*(*A_2_*) (0.784) of “MF” is much larger than the other two probabilities *ṁ^t^*(*A_1_*) (0.193) and *ṁ^t^*(*A_3_*) (0.026). Hence, the driver’s fatigue state can be determined based on the proposed decision rule in Equation (6).

For the 4th sample data point, $y_{1,2}^{t}$ has the maximum 0.488 in the output of T-SFNN-1, and the driver’s fatigue state should be recognized as “MF”. However, as $y_{2,1}^{t}$ in T-SFNN-2 has the maximum 0.721, the driver’s fatigue state will be recognized as “NF”, which conflicts with the result of T-SFNN-1. By contrast, the first evidence fusion result in Table 7 indicates that the driver’s fatigue state is “NF” because *ṁ^t^*(*A_1_*) (0.708) is larger than *ṁ^t^*(*A_2_*) and *ṁ^t^*(*A_3_*). The proposed comprehensive assessment shows that driver’s actual fatigue state is “NF”, which is consistent with the first evidence fusion result. Using video data analysis, we find that the reason why T-SFNN-1 was unable to correctly infer the fatigue state is that the fatigue feature ECD was incorrectly measured due to the driver’s sudden nodding.

For the 5th sample data point, the recognition results of T-SFNN-1 and T-SFNN-2 are also different. One is “SF” while the other is “MF”. By checking the video records, we found that the fatigue feature FADL was incorrectly measured due to the blurred lane and dim lighting, which resulted in the failure of T-SFNN-2 recognition. By contrast, the first evidence fusion result can correctly recognize the driver’s fatigue state.

Table 8 summarizes the final results of the proposed model after the decision-level fusion. The performance of the two levels of fusion model is enhanced because the distinction between *m^t^*(*A_1_*), *m^t^*(*A_2_*) and *m^t^*(*A_3_*) is enhanced. For example, for the 1st sample data point, the difference of the maximum *ṁ^t^*(*A_1_*) and the second maximum *ṁ^t^*(*A_2_*) is increased to 0.883 from 0.495 based on the first evidence fusion result. This enhancement makes the recognition of fatigue state more credible. For the 6th sample data point, the driver’s fatigue state is recognized as “MF” according to the first evidence fusion result shown in Table 7. However, the fatigue state of the 6th sample data point is determined as “SF” according to the decision-level fusion result shown in Table 8. The comprehensive assessment proposed verifies that the actual fatigue state is “SF”, which is consistent with the decision-level fusion result. By analyzing the video data, we found that the GPS device did not receive any signal of vehicle position and velocity because the vehicle entered a tunnel, which resulted in a recognition failure of T-SFNN-2. By contrast, the decision-level fusion was able to obtain correct fatigue recognition by considering the fusion result at the previous time step.

**Table 8:** Recognition results of decision-level fusion.

Index	$\text{\{}{\bar{m}}^{t\text{-1}}\text{(}A_{\text{1}}\text{),}{\bar{m}}^{t\text{-1}}\text{(}A_{\text{2}}\text{),}{\bar{m}}^{t\text{-1}}\text{(}A_{\text{3}}\text{)\}}$	$\text{\{}{\bar{\dot{m}}}^{t}\text{(}A_{\text{1}}\text{),}{\bar{\dot{m}}}^{t}\text{(}A_{\text{2}}\text{),}{\bar{\dot{m}}}^{t}\text{(}A_{\text{3}}\text{)\}}$	*K*	{*m^t^*(*A_1_*), *m^t^*(*A_2_*), *m^t^*(*A_3_*)}	*s_F_*
1	{0.793, 0.102, 0.105}	{0.665, 0.326, 0.009}	0.44	{0.942, 0.059, 0.002}	NF
2	{0.192, 0.713, 0.095}	{0.192, 0.782, 0.026}	0.304	{0.053, 0.801, 0.004}	MF
3	{0.179, 0.599, 0.222}	{0.198, 0.75, 0.052}	0.503	{0.071, 0.904, 0.023}	MF
4	{0.647, 0.285, 0.068}	{0.709, 0.273, 0.018}	0.463	{0.854, 0.145, 0.002}	NF
5	{0.186, 0.127, 0.687}	{0.308, 0.220, 0.472}	0.591	{0.140, 0.068, 0.793}	SF
6	{0.135, 0.079, 0.786}	{0.082, 0.539, 0.379}	0.608	{0.028, 0.109, 0.760}	SF

Finally, to demonstrate the accuracy and robustness of the proposed model, it is compared with the models based on the single feature, and the single-source fusion from three perspectives: accuracy rate (AR), miss rate (MR), and false alarm rate (FAR). Here, AR = (*N*_0,0_ + *N*_1,1_ + *N*_2,2_)/*N*, FAR = (*N*_0,1_ + *N*_0,2_)/*N*, MR = (*N*_1,0_ + *N*_2,0_)/*N*, where *N* is the total number of the samples, and *N_i,j_* is the number of sample data points recognized as having fatigue state *j* when the actual fatigue state is *i*. The performance of the proposed model is also determined based on two sets of feature measurements: (i) using all of the fatigue features; and (ii) using only the most effective fatigue features (excluding MEOL). The results are summarized in Table 9, and illustrate that the proposed model is better than the other models in terms of AR, MR and FAR. Further, while the number of the fatigue features is reduced through fatigue feature identification, the performance of the model is improved. It indicates that the proposed model can provide more accurate and robust results in real-world applications.

**Table 9:** Performance comparisons of five models.

Models	AR	MR	FAR
Single feature based (BF)	88.7%	4.2%	3.9%
Single-source fusion based (Vehicle behavior features and T-SFNN)	90.8%	3.6%	4.1%
Single-source fusion based (Facial features and T-SFNN)	91.6%	3.4%	3.7%
The proposed model (Using all fatigue features)	92.1%	3.1%	3.5%
The proposed model (Based on the most effective features)	93.8%	2.3%	2.8%

Through these analyses, we conclude that the proposed self-adaptive dynamic recognition model with two levels of fusion is effective and robust, even when certain fatigue features become ineffective or some sensors fail because of complex travel environment. In addition, the use of the most effective features can further improve the performance of the proposed model.

## 5. Conclusions

To enhance the effectiveness and robustness of fatigue driving recognition, a self-adaptive dynamic recognition model based on multi-source information and two levels of fusion is proposed in this paper. The accuracy and robustness of fatigue driving recognition are improved through the feature-level fusion as well as the decision-level fusion. The feature-level fusion based on T-SFNN can provide an accurate dynamic probability assignment for the decision-level fusion, while the decision-level fusion based on D-SET is able to adaptively solve the decision conflict caused by external disturbances via combining three pieces of evidence from three different information sources. In addition, the proposed fatigue recognition model is calibrated and verified using a comprehensive assessment method of the fatigue state and the data collected from field experiments.

The experiment results demonstrate that the proposed model performs well in terms of accommodating the disturbances caused by complex environment changes. When the most effective fatigue features are selected and applied to the proposed model by conducting a correlation analysis, the performance of the model is further improved in terms of accuracy and reliability. In addition, compared to models based on the single fatigue feature and/or single-source fusion, the proposed fatigue recognition model provides more accurate and robust results in terms of the accuracy rate, missing rate, and false alarm rate. Therefore, the proposed fatigue recognition model can perform better in real-world applications to improve travel safety.
